# A *de novo* variant in the bovine *ADAMTSL4* gene in an Original Braunvieh calf with congenital cataract

**DOI:** 10.1111/age.13178

**Published:** 2022-03-01

**Authors:** Irene M. Häfliger, Sonja Wolf‐Hofstetter, Christina Casola, Udo Hetzel, Franz R. Seefried, Cord Drögemüller

**Affiliations:** ^1^ Institute of Genetics Vetsuisse Faculty University of Bern Bern Switzerland; ^2^ Ophthalmology Section Equine Department Vetsuisse Faculty University of Zurich Zurich Switzerland; ^3^ Institute of Veterinary Pathology Vetsuisse Faculty University of Zurich Zurich Switzerland; ^4^ Qualitas AG Zug Switzerland

**Keywords:** *Bos taurus*, development, eye, lens, precision medicine, whole‐genome sequencing

## Abstract

Inherited forms of cataract are a heterogeneous group of eye disorders known in livestock species. Clinicopathological analysis of a single case of impaired vision in a newborn Original Braunvieh calf revealed nuclear cataract. Whole‐genome sequencing of the parent‐offspring trio revealed a de novo mutation of *ADAMTSL4* in this case. The heterozygous p.Arg776His missense variant affects a conserved residue of the *ADAMTSL4* gene that encodes a secreted glycoprotein expressed in the lens throughout embryonic development. In humans, *ADAMTSL4* genetic variants cause recessively inherited forms of subluxation of the lens. Given that *ADAMTSL4* is a functional candidate gene for inherited disorders of the lens, we suggest that heterozygosity for the identified missense variant may have caused the congenital cataract in the affected calf. Cattle populations should be monitored for unexplained cataract cases, with subsequent DNA sequencing a hypothesized pathogenic effect of heterozygous *ADAMTSL4* variants could be confirmed.

Congenital ocular conditions such as cataract causing impaired vision or complete blindness have been documented in cattle (Williams, 2010). It was established more than 100 years ago that cataracts in mammals can be environmental or hereditary (Detlefson & Yapp, 1920). For example, congenital ocular lesions including primarily cortical cataracts have been reported in calves exposed to bovine viral diarrhea virus in utero (Bistner et al., 1970; Siepker et al., 2021). However, often no definitive nutritional, infectious, or toxic cause for the congenital cataract is identified in affected herds (Krump et al., 2014; Osinchuk et al., 2017). Sporadic occurrence of inherited forms of cataract in certain breeds of cattle has been reported (OMIA000168‐9913). Detlefson and Yapp (1920) reported evidence of monogenic autosomal recessive inheritance in Holsteins and Gregory et al. (1943) reported equally convincing data in Jerseys. Nonetheless, so far, causative variants were found only for two rare breed‐specific recessive forms of bovine cataract: the *NID1*‐related nuclear cataract of Romagnolas (OMIA001936‐9913; Murgiano et al., 2014), and the *CPAMD8*‐related Morgagnian cataract of Holsteins (OMIA002111‐9913; Hollmann et al., 2017). Such findings enable selection against these disorders within the affected populations. Furthermore, the cataract observed in Romagnolas was the first report of a naturally occurring mutation of *NID1* (Murgiano et al., 2014). This led to a non‐syndromic form of cataract in a mammalian species and added the affected gene to the list of candidate genes for inherited forms of nuclear cataract in humans (Murgiano et al., 2014). In human medicine, about 50% of cataracts are thought to be associated with genetic factors and causative variants for congenital or other early‐onset forms of cataracts have been discovered in over 30 genes (Shiels & Hejtmancik, 2017). Congenital cataract has also been shown to be a heterogeneous group of diseases in cattle, as the reported *CPAMD8* nonsense variant is obviously not sufficient to explain the majority of Holsteins suffering from Morgagnian congenital cataract (Braun et al., 2019).

In the present study, we investigated a 1.5‐month‐old male Original Braunvieh calf with clinical signs of cataract showing bilateral opaque eye lenses (Figure 1). According to the owner, the cataract had been present since birth. At first, the calf had problems finding the teats to suckle the milk and initially ran into walls and objects, but after a few days, it was able to orientate itself. Clinical examination revealed no further abnormalities and the calf was referred to an ophthalmologist. The calf presented to the ophthalmology service with open eyes. Dazzle and pupillary light reflexes were normal, but menace response was absent in both eyes. Intraocular pressure was within normal limits (right eye 20 mmHg, left eye 19 mmHg). The lens showed in both eyes a diffuse dense nuclear opacity and a focal axial anterior cortical opacity in addition with multifocal anterior capsular punctual opacities. Fundic examination was mostly blocked by lens opacities, the visible parts in the periphery were normal. Further ophthalmic examination showed no more abnormalities and the calf was diagnosed with an immature cataract in both eyes. The parents of the affected calf were clinically normal. At the time of slaughtering, the calf was 4.5 months old and appeared in a good general health condition.

**Figure 1:**
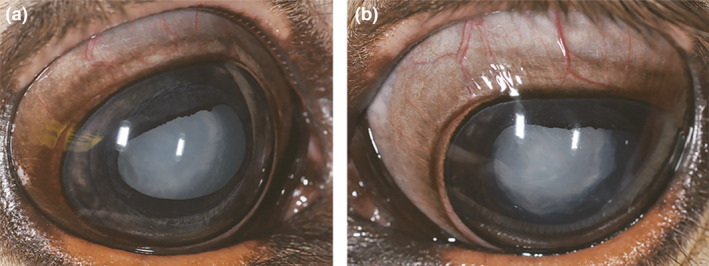
Original Braunvieh calf with congenital cataract. Opaque lenses of right (a) and left (b) eye are shown at the ophthalmological examination at 1.5 months

Macroscopically, the regularly shaped ocular bulbs demonstrated normal sized lenses with moderately diffuse clouding with clear demarcation of the capsules and regularly developed zonula fibers (Figure 2a,b,d). Histologically, the anterior half of lental protein appears normal, whereas from the equator onwards posteriorly, a severe lental nuclear cataract composed of eosinophilic partly fibrillary, partly amorphous lental proteinaceous material with multifocal small foci of mineralisation (calcification) is present (Figure 2c,e,f). The anterior lens capsule is well developed, the anterior epithelium and equator show a regular formation whereas the posterior lens capsule reveals a moderate fibrillation (Figure 2g, right side). No evidence for infectious agents could be observed in hematoxylin and eosin as well as special stains. All other intraocular structures appeared histologically normal. The bovine embryonal lental development occurs during the first trimester of gestation with formation of the lens placode to development of the lens vesicle between 3.3‐ and 14‐mm embryonal crown rump length (Schnorr & Kressin, 2006). Lenses show continuous lifelong growth with strongest growth rate during embryonal and morphologically non‐altered development and the 1st year of life (Levin et al., 2011). The lenses presented here are fully developed with an intact lental capsule, zonula fibers, and regularly arranged and morphologically normal equatorial cells. Thus the opacification of the lental protein, i.e. the formation of a nuclear and posterior subcapsular cataract, must have developed in a relative late stage of the lental development, morphologically widely comparable to the findings demonstrated in the *NID1*‐related nuclear cataract of Romagnolas (Murgiano et al., 2014) and Morgagnian cataracts in Red Holstein Friesian cattle (Braun et al., 2019).

**Figure 2:**
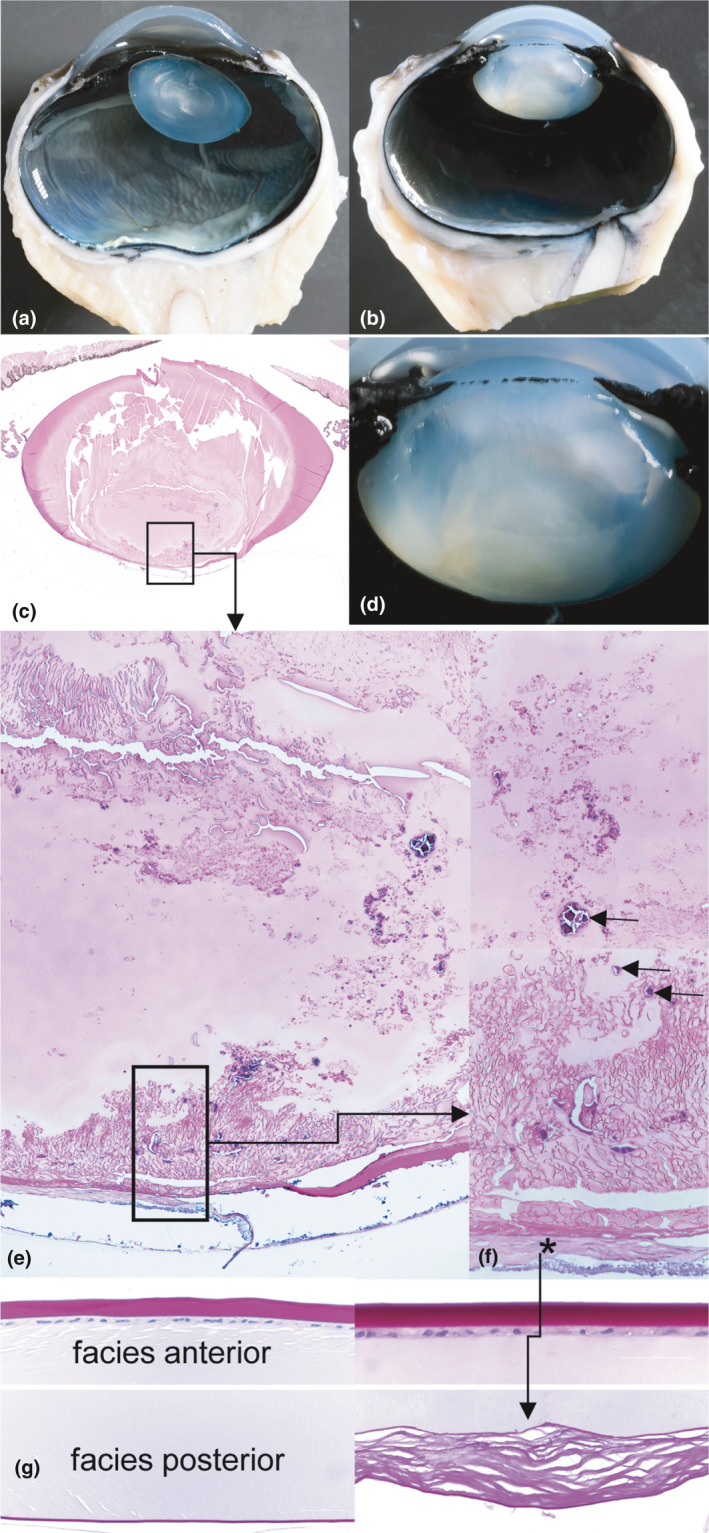
Features of nuclear cataract in an Original Braunvieh calf. Normal eye, sagittal section (a). Altered right eye with severe lental nuclear cataract (rectangle), sagittal section (b). Histological overview (c) of the cataractous lens depicted in (b). Higher magnification (d) of the lens depicted in (b). Facies posterior of the lens with cloudy appearance of lental protein and multiple foci of mineralization (e). Higher magnification of the posterior lens capsule, with perilental collagenous membrane (asterisk) and fibrillar lental protein with mineralization (arrows) (f). Comparison of the anterior and posterior lens capsule, left side: normal bovine lens, right: altered fibrillary posterior capsule (g)

We prepared a PCR‐free DNA library of the affected calf and its dam and collected short read pairs (2 × 150 bp) to obtain roughly 25× coverage on an Illumina NovaSeq 6000 instrument. The whole genome sequencing data of the sire was publicly available . Variants in the genome of the affected calf were called with respect to the reference genome assembly ARS‐UCD1.2 as described previously (Häfliger et al., 2020). A hard filtering approach was applied by comparing the variants to a cohort of 5115 cattle genomes containing both parents. Given that the mode of inheritance was unknown and assuming that the causative variant is rare, we filtered for variants that were only present in the affected calf, either in heterozygous or homozygous state. All 5116 genomes are included in the variant catalogue of run 9 of the 1000 Bull Genomes Project including animals of more than 130 genetically diverse breeds (Hayes & Daetwyler, 2019). Assuming recessive inheritance, filtering for coding variants present only homozygous in the calf and heterozygous in the parental genomes identified no coding variant. Assuming a dominant mutation, filtering revealed a single private protein changing variant, a missense variant in the *ADAMTSL4* gene (NM_001101061.1:c.2327G>A, p.Arg776His), with a heterozygous genotype in the affected calf that was not detected in any of the control genomes including both parents. This C‐to‐T transition at position 20146737 on chromosome 3 located in exon 12 of *ADAMTSL4* is predicted to alter the encoded amino acid of ADAMTSL4 residue 776 (NP_001094531.1:p.Arg776His) to histidine, a less polar and more hydrophobic amino acid than arginine. The affected residue represents a probably functionally important and conserved residue located in the third of seven thrombospondin type 1 repeat domains (Figure 3). Although the arginine to histidine substitution was predicted to be neutral using PROVEAN software (Choi & Chan, 2015), it remains unclear whether this amino acid substitution affects protein folding or function. Sanger sequencing confirmed the mutant allele to be present in a heterozygous state in the affected calf and absent from its parents, given that the variant was not found in the paternal germline DNA analyzed (Figure 3). This means that the c.2327G>A variant probably arose *de novo* spontaneously during very early development of the calf.

**Figure 3:**
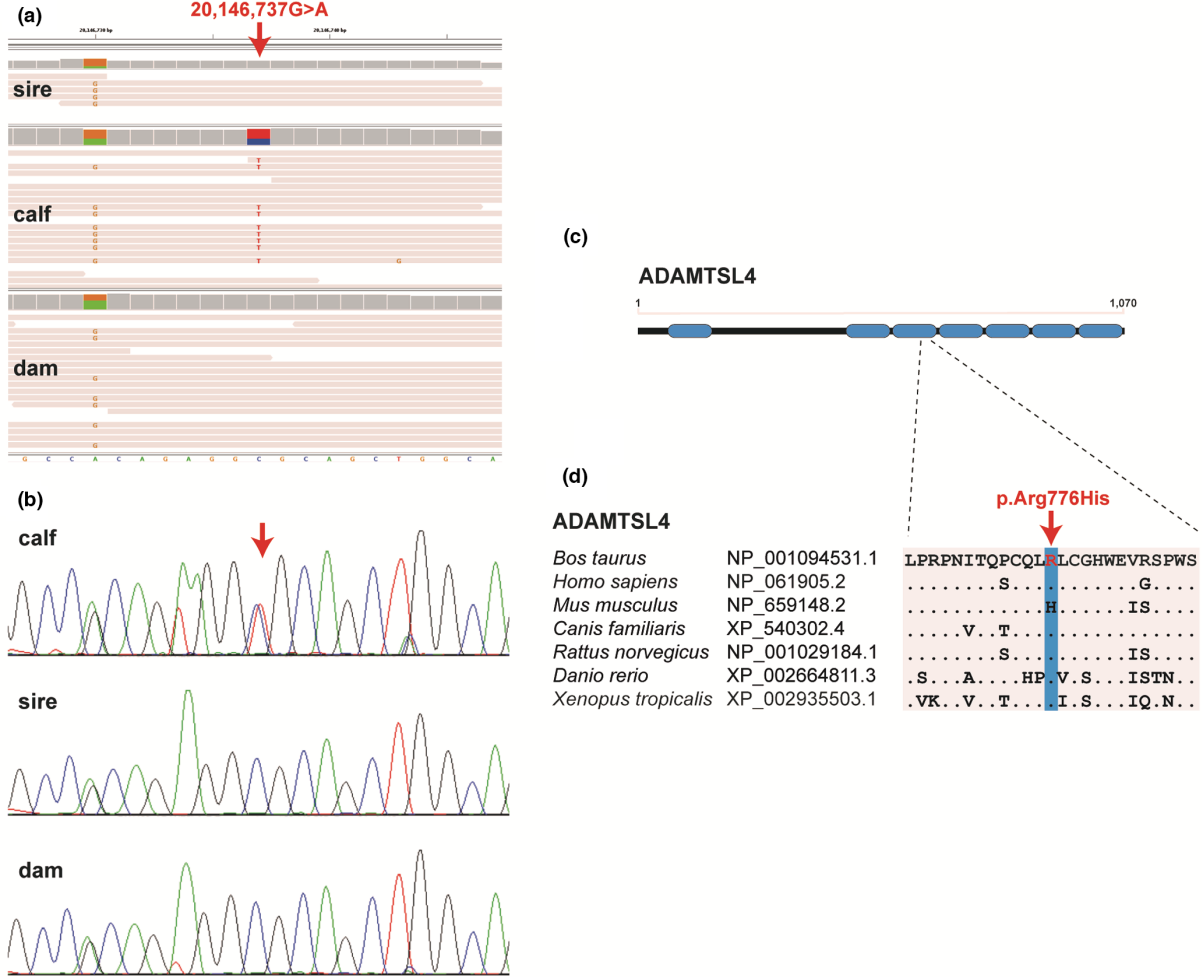
Details of the detected *ADAMTSL4* variant. (a) Integrative Genomics Viewer screenshot presenting the heterozygous single nucleotide variant (red arrow) present only in the affected calf. (b) Sanger sequencing results confirmed that the variant occurred *de novo* as sequencing of PCR products from DNA of both parents (for the sire semen) showed that the c.2327G>A variant was absent. (c) Schematic diagram of the bovine ADAMTSL4 protein that has a repetitive domain structure with seven thrombospondin type 1 repeat domains (blue). The p.Arg776His variant (red arrow) affects the third repeat. (d) Multiple species amino acid alignments encompassing the region of the variant demonstrates a high evolutionary conservation across species. The observed variant is indicated by an arrow and the respective position highlighted in gray

The formal possibility exists, however, that the detected protein‐changing *de novo* mutation is simply a functionally neutral change, but we regard this possibility as unlikely because of the following reasons. Various ADAMTS (a disintegrin‐like and metalloproteinase with thrombospondin type 1 motifs) proteins are necessary for normal ocular development and eye function (Mead & Apte, 2018). ADAMTS‐like proteins lack a metalloprotease domain, reside in the extracellular matrix and have regulatory roles and ADAMTSL4 has been implicated in fibrillin microfibril biogenesis (Gabriel et al., 2012). *ADAMTSL4* is widely expressed in non‐ocular tissues as well as in various eye components, particularly in the lens equatorial epithelium when the zonule attaches (Chandra et al., 2013; Gabriel et al., 2012). Besides focal retinal pigment epithelium defects, homozygous disruption of murine *Adamtsl4* resulted in a defect in the anchoring of zonule fibers to the lens surface, causing ectopia lentis, confirming its role in zonule formation (Collin et al., 2015). In humans, recessive isolated ectopia lentis (subluxation or dislocation of the human crystalline lens) and ectopia lentis et pupillae are caused by *ADAMTSL4* loss‐of‐functions variants (OMIM610113). Heterozygous carriers of the known nonsense or splice site variants were reported to be apparently normal probably due to nonsense‐mediated decay of the aberrant transcripts. Finally, visual inspection for large structural variants in the genome, performed after plotting the average read depth across the entire genome for the sequenced trio, revealed no obvious evidence of this type of mutation either at the chromosomal level or in the region of the *ADAMTSL4* gene (Figure S1).

Therefore, we propose the c.2327G>A variant as candidate causative variant for the observed congenital cataract phenotype based on the following arguments: (1) given that only one protein‐changing *de novo* mutation event per generation is expected on average (Heinzen et al., 2015), this strongly supports the causality of the variant; (2) evolutionary conservation and expansion of ADAMTSL proteins in mammals indicates a crucial role in embryonic development (Mead & Apte, 2018); (3) in mice, it was shown that *ADAMTSL4* is strongly expressed in the lens epithelium at the lens equator throughout embryonic development (Collin et al., 2015); and (4) patients with *ADAMTSL4*‐related ectopia lentis commonly present with a marked loss in visual acuity in addition to a number of possibly accompanying ocular complications including early cataract development (Ahram et al., 2009; Christensen et al., 2010). Given that this is a single case investigation and that we have no functional confirmation, this result must be considered preliminary and should be interpreted with caution. However, it must also be emphasized that the analysis was not suitable for identifying larger structural variants. Further isolated cases of cataract in cattle could be investigated for *ADAMTSL4* variants by DNA sequencing.

## CONFLICT OF INTEREST

The authors declare that they have no competing interests.

## Supporting information

Fig S1

## Data Availability

The whole genome sequencing data of the animals generated in this study is publicly available at ENA project accession PRJEB18113 (https://www.ebi.ac.uk/ena/browser/view/PRJEB18113) with sample accessions SAMEA5159837 (case) and SAMEA6528886 (dam). The whole genome sequencing data of the sire was publicly available (ENA project accession PRJEB28191 (https://www.ebi.ac.uk/ena/browser/view/PRJEB28191), sample accession SAMEA5059753).
