# Supplementary material for: A de novo variant in the bovine ADAMTSL4 gene in an Original Braunvieh calf with congenital cataract
Source: Anim Genet. 2022 Mar 1;53(3):416–21. doi: 10.1111/age.13178 (PMC9311076; doi:10.1111/age.13178)
Supplement: Supplementary file 1 — Fig S1 [file AGE-53-416-s001.pdf]

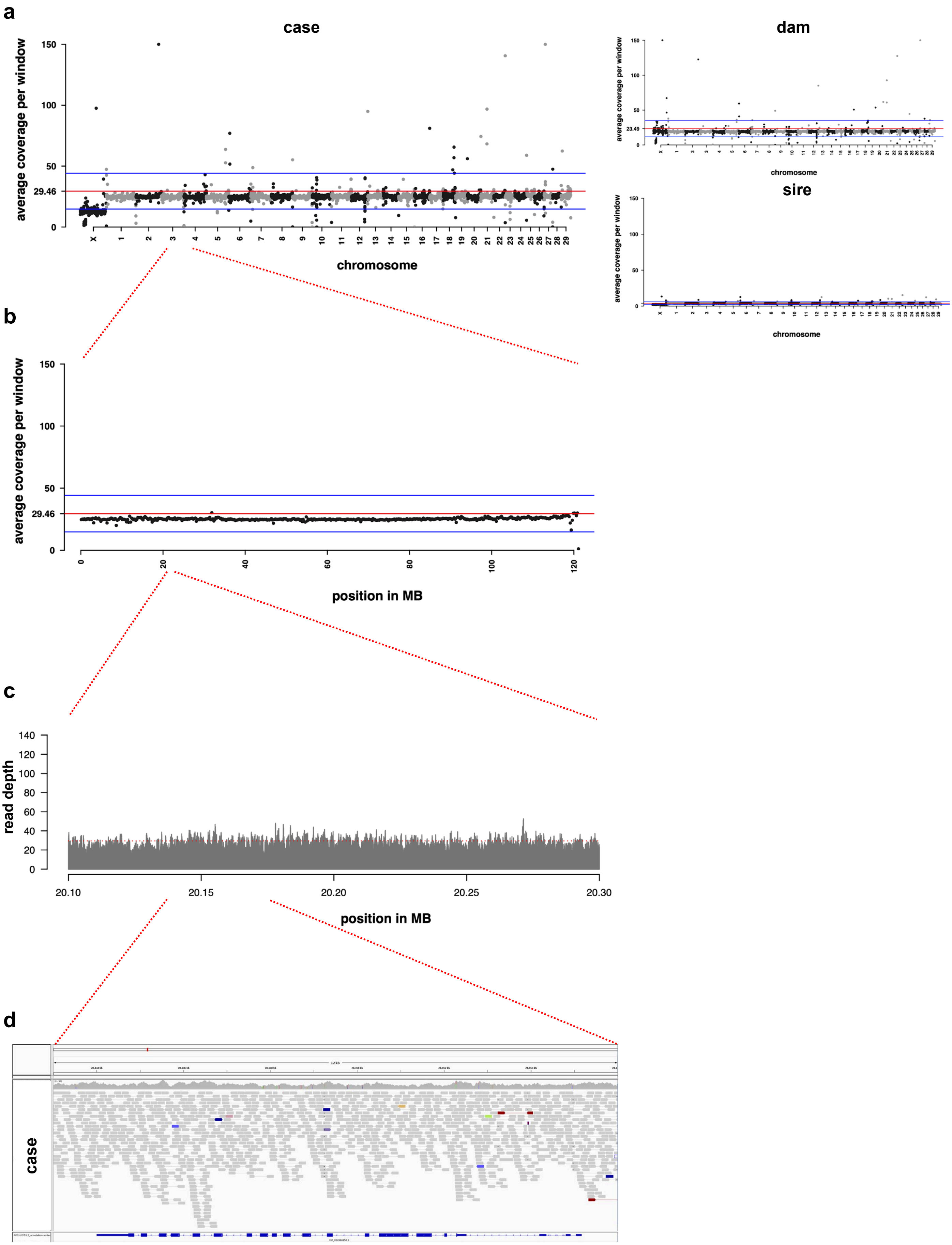

Figure S1 Visual inspection for large structural variants in the genome of the affected calf. Read depth of 250kb-wide windows across the genomes of the sequenced trio (a), and across chromosome 3 for the case (b). Note that no chromosomal aberrations are detectable. Read depth per base surrounding *ADAMTSL4* (c) and IGV screenshot of the entire gene region (d), indicating no systematically misaligned reads in the genome of the sequenced case.
